# A biomimetic study of natural attachment mechanisms: imaging cellulose and chitin part 2

**DOI:** 10.1186/s40638-015-0032-9

**Published:** 2015-12-09

**Authors:** Bruce E. Saunders

**Affiliations:** University of Bath, 54 Ballance Street, Bath, UK

**Keywords:** Reverse engineering, Shape acquisition, Confocal microscopy, Rapid prototyping, Cellulose, Insect chitin, Scaling effects, Miniature, Micro-mechanisms, Biomimetics

## Abstract

To create a model of a biomimetic product from the *A. minus* hook after a biomimetic methodology has been applied, this paper describes an investigation into the most appropriate method of shape acquisition for the purposes of reproduction and product development towards manufacture. This morphological study investigates confocal microscopy, SEM and other microscopy techniques. Confocal microscopy was selected as being most appropriate and small structures of cellulose and insect cuticle imaged. The benefits and disadvantages of this approach are noted. This paper is the result of research into microscopy techniques coupled with state-of-the-art manufacturing techniques. The result is this experiment with a single-phase confocal microscope to capture their 3-D images, both of cellulose and of chitin, without any specimen-specific treatment. Emphasis must be placed upon the cleanliness of the process since so many Natural attachment mechanisms are of this order of size and confocal microscopy offers opportunities for physical examination of microstructures and their interaction, in situ, with non-destructive inspection. This methodology has to develop further for the source of Nature’s designs to be rifled for ideas.

## Background

This project received funding from the EPSRC under the title: “The Functional Ecology and Mechanical Properties of Biological Hooks”, a wide field that was subsequently refined to three research papers.

The first part of the series [1] explores the methodology of the biomimetic process and refines it further, producing an accurate model for the static loading case that indicates scaling effects are present in a cellulose hooked-structures of span 200 μm or less.

The study now proceeds to the broader case of studying all Natural Hooks and the available technology for examining these small (~200 μm) structures with new eyes and the opportunities they present for modelling, stress analysis and virtual reality. Confocal microscopy, in particular single-phase confocal microscopy, comes to be the technique of choice and is used here in this part, the second research paper, to examine the structural, frictional and other types of probabilistic attachment mechanisms that are the burdock hook and the tarsal structures of two insects, a common honey bee and a common grasshopper, to Great Britain.

There is much in the literature about many aspects of this study, from studies of the biomaterials [2] to studies of the insect structures themselves by Gorb and Betz [3, 4] that it seems arrogant to expect to isolate some aspect by fortunate chance that proves of biomimetic importance. However, it is believed that it is not a variety of structures that is required, but only a single example to be adequate, and that many structures have not been studied for use in transfer of function thus far [5]. Therefore, a field of specimens of varying aspects/characteristics needs to be modelled and their performance compared. Within the literature, however, consideration is only given to how the attachment structures act within their ecological systems. For example, a tarsal structure of an insect is not considered to be a model for a robotic handling device: it is considered as a part of the locomotor systems of the insect/organism. Studying these structures with the aid of modern computing and layered manufacture and microscopy techniques with ambitions to cross-pollinate with technology seems the answer and that is the fundamental notion underpinning this paper.

This work was done in September 2003. At that time rapid prototyping and layered manufacture were in their infancy. Ill health lead to a delay in the release of the results and a destruction of all relationship with the Institution (the University of Bath) where the research took place and subsequently the Centre for Biomimetics and Natural Technologies was shut down in 2008, preventing me from re-joining the group and publishing.

## Introduction: morphological studies

This phase of the study concerns itself with recording 100-micron range hook shapes for the purposes of engineering analysis and reproduction, looking at the nature of the outputs of various microscopy techniques and their transferability to the domain of manufacturing. The aims of [1], its conclusions and its ambitions towards an overall aim of describing a process for the design of micro-sized structures and sub-structures as friction devices as required in microsurgical devices and computer hardware are used as a case study. The design of such structures must be influenced by scaling factors and, therefore, unexpected non-Newtonian quantities. However, it is envisaged that as a first approximation of behaviour, to imitate structures found in nature from nano/self-assembled biomaterials may be a suitable starting point for design. The problem with most current microscopy vision systems is their output is not suitable for virtual reality. If they are, then they are not suitable for FEA or layered manufacture for the purposes of analysis and modelling because their file formats are incompatible with.stl.

The functional ecology of a system is the forces that are exerted within that system and their interactions and how they influence the property being studied. In the case of being manufactured/formed/grown it is the case that harvesting hooks may be a better idea than manufacturing them. Cladistically speaking it makes sense that there should be some join between the cellulose and chitin that does not exist in Nature, but it was found that Archeopteryx had feathers and scales so this does not preclude a future discover that mounts the shape of things to come as being brown and chitin-esque. All the more for a procedure that generates an impeller to bring these secrets to light.

The scaling effect observed in [1], namely the reduction in the influence of the span of the hook on its tensile strength, is to be observed in any forthcoming design.

It has yet to be heard of, an engineer designing in artificial cellulose or chitin and this is accepted as a fact, because of the severe limitations of the reach of technological progress. However, as a study that produces insight into both surface strata of attachment as well as methods of study thereupon, it is though it retains its import whether years old or not.

### Shape acquisition

Shape acquisition has a history in biological studies. From the first cave drawings man has endeavoured to reproduce that which he observes in nature. Today, shape is used to provide clues as to internal composition of a biological structure when considered in the context of biomaterial strengths and behavior and the use of the principle of shape optimization.

Dai, Gorb and Schwarz [3] used methods of analysing 2-D radii of curvature in insect tarsi to identify structural anomalies which superficially would seem to indicate zones of weakness or stress concentration but in reality identify zones of localised hardening/strengthening due to the presence of zinc or other trace minerals in the insect cuticle. When a structure does not break under loading when the shape of the structure would seem to indicate that it should, there is an indication that some material discontinuity is responsible.

Beraldin et al. [6] in their paper on the virtual reality applications of scanning technology discuss the use of data transfer for layered manufacture and rapid prototyping. Confocal microscopy makes use of light intensities provided by fluorescing molecules to form images of minute structures and their internal components. Evans et al. [7] used this physical phenomenon and technology, by casting the external morphologies of bat’s teeth to generate 3-D images of teeth to study wear patterns.

Finite element analysis comprises the precise division of a 3-D morphology into vertices and edges to compute stresses at a distance from an applied load in a structure. It was judged that to be of most use, the output file format from the microscopy/imaging technique would allow for both computer vision and finite element analysis as well as rapid prototyping and so the investigation began for a microscopy technique that support this ambition for the purposes of study of microsurgical instrumentation and other probabilistic and specific fasteners in particular of varying textures.

## Aim

The above introduction prompted the following questions:

1. What forms were the outputs of the available microscopy techniques and did they lend themselves to virtual reality/mechanical analysis?
2. Can small structures be imaged using a confocal microscope without the use of the casting methods of Evans et al.?
3. With reference to confocal microscopy output, can a mesh formed from random points of light intensities be used to form a finite element analysis mesh? Since light intensities are the recorded outputs of the sensors in the microscope, a voxel.
4. Can a mesh formed from random points of light intensities be used to form a mesh for conversion to.stl format and sent to a rapid prototyping device?

### Apparatus

#### Microscopy techniques

The following techniques were the focus of preliminary investigation, through the lectures of Dr I Jones, then of the Neuroscience Department at the University of Bath [5]:

1. The principles of fluorescence microscopy.
2. Epi-fluorescence microscopy.
3. Confocal microscopy.
4. 2-photon microscopy.
5. Near-field scanning optical microscopy.

The fluorescence effect is produced by irradiating atoms with a high-energy light source (laser) which causes excitation of orbiting electrons. These electrons jump “outwards” to high-energy orbitals before returning to their normal state, releasing energy at a specific wavelength which is detected via an emission filter.

Confocal microscopy makes use of a laser light source whereas epi-fluorescence microscopy makes use of a normal bright light source and two filters, an excitation filter and an emission filter. Samples are viewed through an eye-piece.

The advantages of confocal microscopy using a laser light are the following:

- reduced blurring;
- increased effective resolution;
- improved signal to noise ratio;
- z-axis scanning;
- depth perception;
- magnification is electronically adjusted;
- scope for clear/good examination of relatively (~100 μm) thick specimens.

From a mechanical engineering/manufacturing perspective it is the output of the confocal microscope that makes it most apparently attractive, voxels and.tif files, as well as the huge convenience as a data acquisition source, using simply mounted slides. Stacked an image is produced in.avi format ready for virtual reality applications.

The following procedure is described by Evans et al. for the production of cubic voxels and virtual reality applications from his paper on the imaging of mammalian teeth [4]. It notes how to take a suitable image of a small object (~mm) for the purposes of digitising, reproduction and study in virtual reality.

In capturing an image it must be borne in mind that the goal was an accurate 3-D model for both virtual reality applications. It is important to set the slice thickness accordingly to arrive at an undistorted image, i.e. cubic voxels. The paper by Evans et al. details a method of taking a cast of a tooth which is more technically cumbersome than simply putting a microscope slide with specimen under the objective and so some of his paper is not relevant here (the details concerning the casting of the teeth).

Optical slices were taken through the *x, y* plane where each slice was square (e.g. 256 × 256) pixel 8-bit image at medium scanning speed.

Slices must be taken at the same distance as the interval between pixels to make cubic voxels.

Software such as Zeiss is used to generate a 3-D image from the stack of slices, where pixel intensity represents height and the z-height is found by comparing the intensities for each *x, y* point (in fact, a column of pixels all with co-ordinates (*x*, *y*)). In most of the tests run by Evans et al., the cubic voxels (and *z*-interval) were 7.8 μm long, generated using one of two following methods:

1. For the ×5 mm lens—a 256 × 256 pixel image was scanned at zoom 1 (field of view (FOV) of 2 × 2 mm), or a 128 × 128 pixel image was scanned at zoom 2 (FOV 1 × 1 mm).
2. For the ×10 mm lens, a 128 × 128 pixel image was scanned at zoom 1 (FOV 1 × 1 mm).

Therefore using a lens with a field of view (FOV) of 2 × 2 mm at a setting of zoom 2 reduces the field of view to 1 × 1.

Surface noise can affect the image and give a false indication of where the true surface lies. Evans et al. did experiments with the ×5 and ×10 lens to see how best to obtain the most accurate surface image. They used two techniques to try to reduce surface noise; *accumulation* and *averaging*. Accumulation is to accumulate and average several images at each *z* height and then create an image from the accumulated image slices. On the microscope, for example, an “Accumulation 2” scan stands for the number of slices that are averaged (two).

The second method was to take the average of a number of reconstructed 3-D images of the same area. This was tested using a specially prepared and dimensionally precise standard glass specimen and comparing resultant images. The specimen was cubic and so without any undercuts but with a 45° fillet. the inner width was 1.3 mm and outer width 1.7 mm.

It was found that averaging produces better results than accumulation. Sanson et al. used a resin casting of their teeth specimens which was coated with eosin, a fluorescent dye. Sanson et al. used their generated model to study the interactions of teeth, not for stress analysis or reverse engineering purposes.

### Confocal microscopy: apparatus and method

Conventionally confocal microscopy is used in neuroscience to study cell organelles. It was decided that small biological specimens many times larger than the average organelle could possibly be translucent enough to laser light such that it might not be necessary to use the casting method of Evans et al. Instead it was decided to attempt to image plain untreated specimens of cellulose and insect chitin. This seemed a rational cross-pollination of technology. The first step was to mount the specimen and the simplest possible well-mount in distilled water was used.

A specimen *A. minus* bract was mounted upon a “well” microscope slide in distilled water (it is a feature of both confocal and atomic force microscopy that specimens may be mounted without treatment) and placed under the objective of a confocal microscope.

### Confocal microscope and scanner

- A Zeiss Axiovert single photon confocal microscope (inverted microscope with the objectives underneath the platform).
- Zeiss LSM 510 module (laser scanning microscope) with 2 lasers.
  - 1 × Argon (488 nm).
  - 2 × HeNe (543 and 633 nm).

### Objectives

- all ×10, 40 (oil), 63 (oil) and 63(water).
- digital zoom up to ×200.
- differential interference contrast.

The field of view: 1 × 1 mm.

Pinhole setting: 1 optical unit.

Scanning slice thickness: 19 nm.

Also

- Well slides which are microscope slides with a bowl ground out in the centre to receive specimens that are not flat.
- Distilled water as a medium for slide mounting.
- It is important to get the hooked specimen in the right orientation on the slide to avoid displaying an undercut surface to the laser light.
- It is important to optimise the strength of the laser and reduce the required depth of penetration to prevent excess bleaching.
- The same specimen can be remounted a number of times in different orientations in the slide to fully expose the complete detail of the structure.

## Results

When suffused with the laser light at three different frequencies it was found that the cellulose *A. minus* hook fluoresced well under the green laser light. Under the red and blue light the resulting image was less distinct but these colours worked well for the insect tarsi. The stacked image is then output to file and stored as a sequence of.tif files that are viewed in.avi format (see below for the full range of.tif images).

### Burdock hook stereograms

**Fig. 1:**
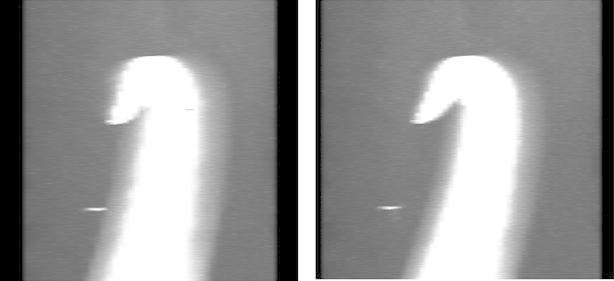
Stereogram 1 of the burdock hook specimen (Dr I Jones, October 2002)

Stereogram images of the hook follow (Fig. 1). The data from the confocal microscope are a sequence of image slices that are then automatically reassembled (stacked). Evidence of the stacking can be observed in the images from the stepped outline of the image. The glow that surrounds the stereogram image derives from the fact that this view of the hook is assembled using standard confocal software and the viewer is looking through preceding and following images which are a result of the perspective of looking at angled images. Only in a profile image does a stark outline of the hook show. There is an artefact on the microscope slide that shows to the side of the hook.

### Burdock .tif images

**Fig. 2:**
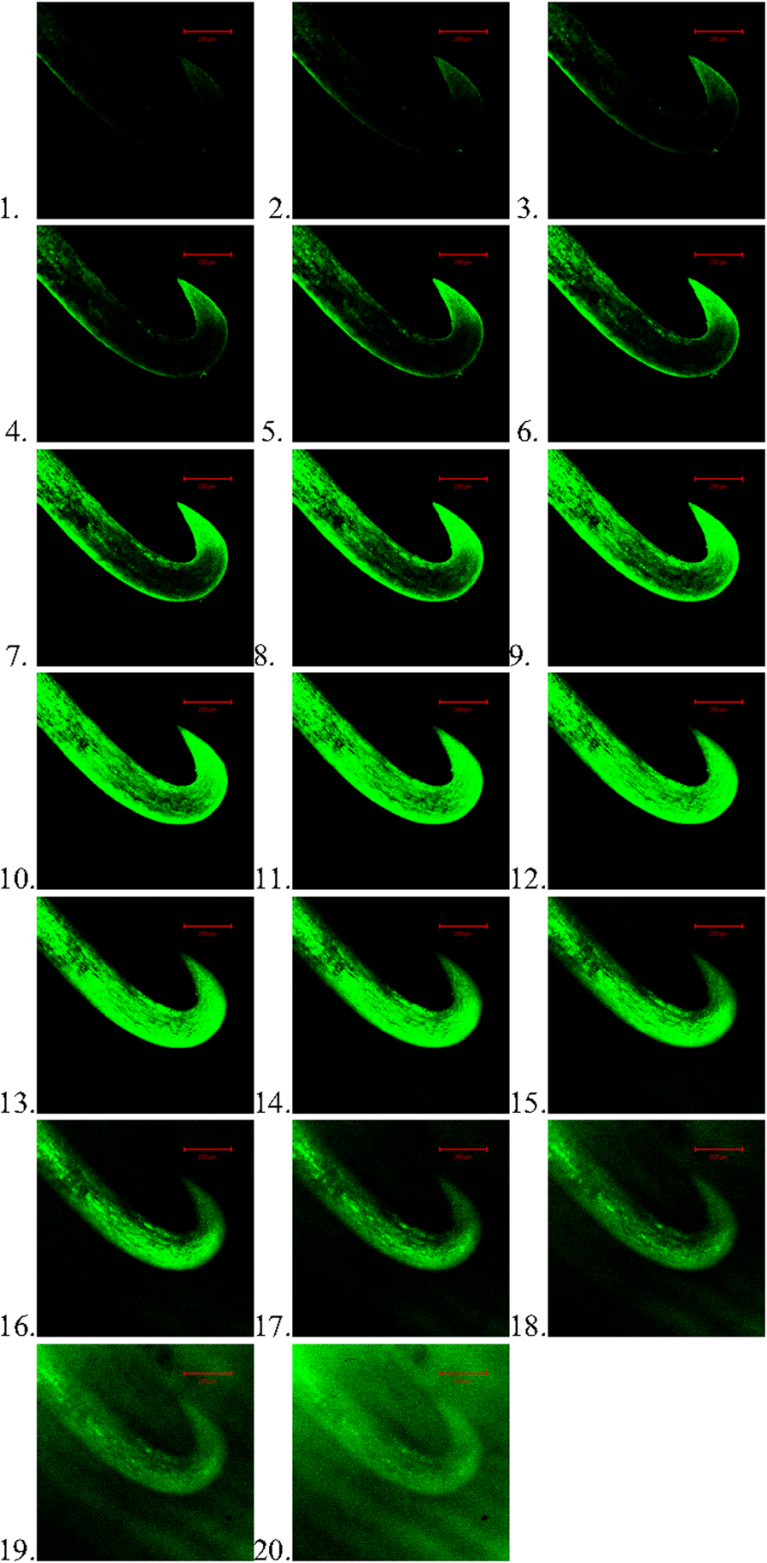
*1*–*20* The individual z-axis scan.tif files that make up the stereogram of the burdock hook (the *scale bar* defines 200 μm. Dr I Jones October 2002)

The individual .tif images that make up the above stereogram below (see Fig. 2) were imaged under the green light. The specimen was placed upon its side for the z-axis scan to minimise the number of scans required to scan the entire specimen, with the hook in profile to take into account undercut of the hook.

Note that the images from the confocal microscope show some internal structure of the hook, particularly the cellulose microfibrils. These microfibrils were visible in [1] which fractured the hooks in a tensile tester. The hooks are made up of cellulose fibres bound together with hemi-cellulose to form microfibrils [6].

**Fig. 3:**
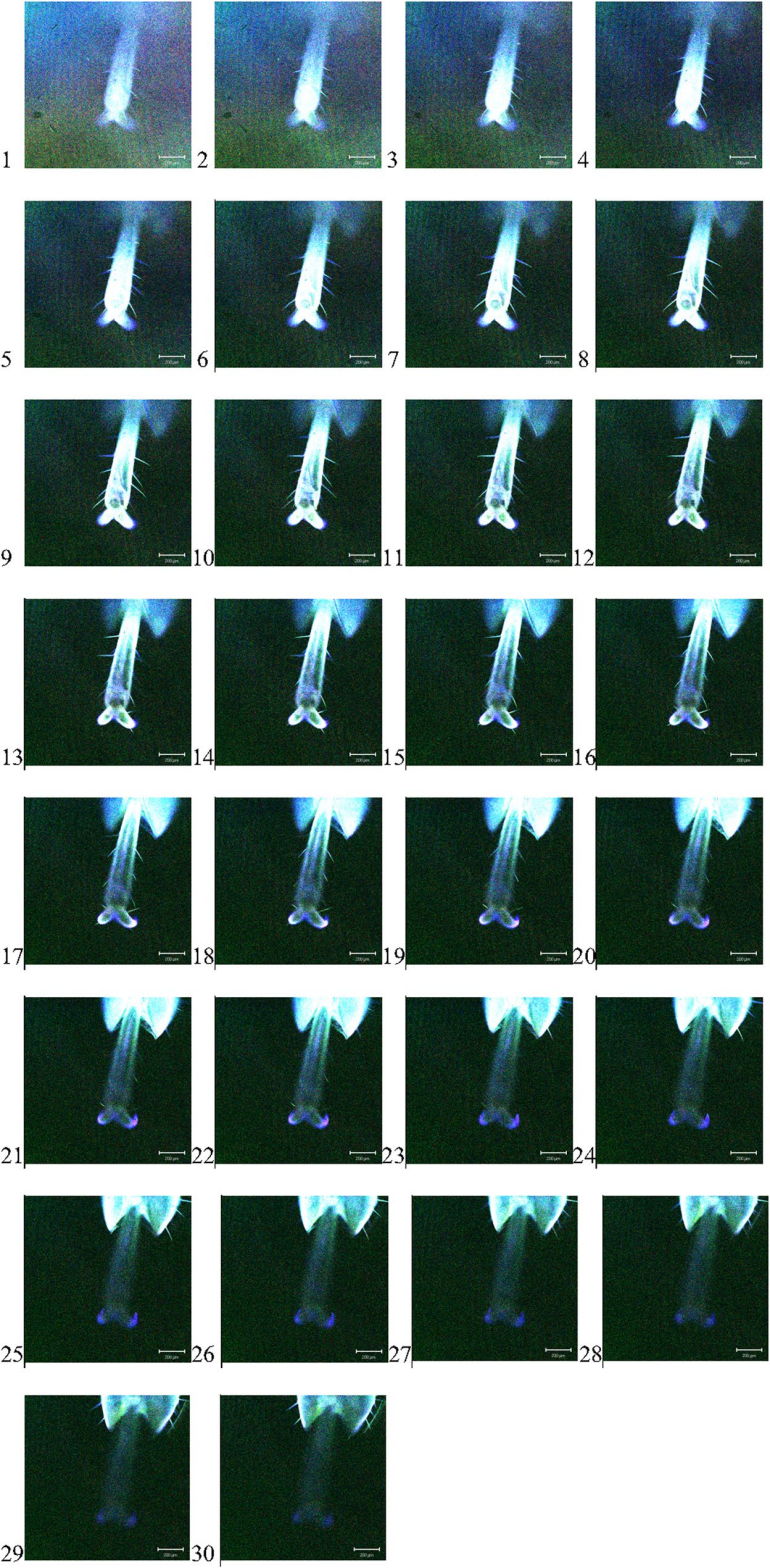
*1*–*30* The individual z-axis scan .tif files of the scan through the tarsus of a common grasshopper (the *scale bar* defines 200 μm. Dr I Jones October 2002)

**Fig. 4:**
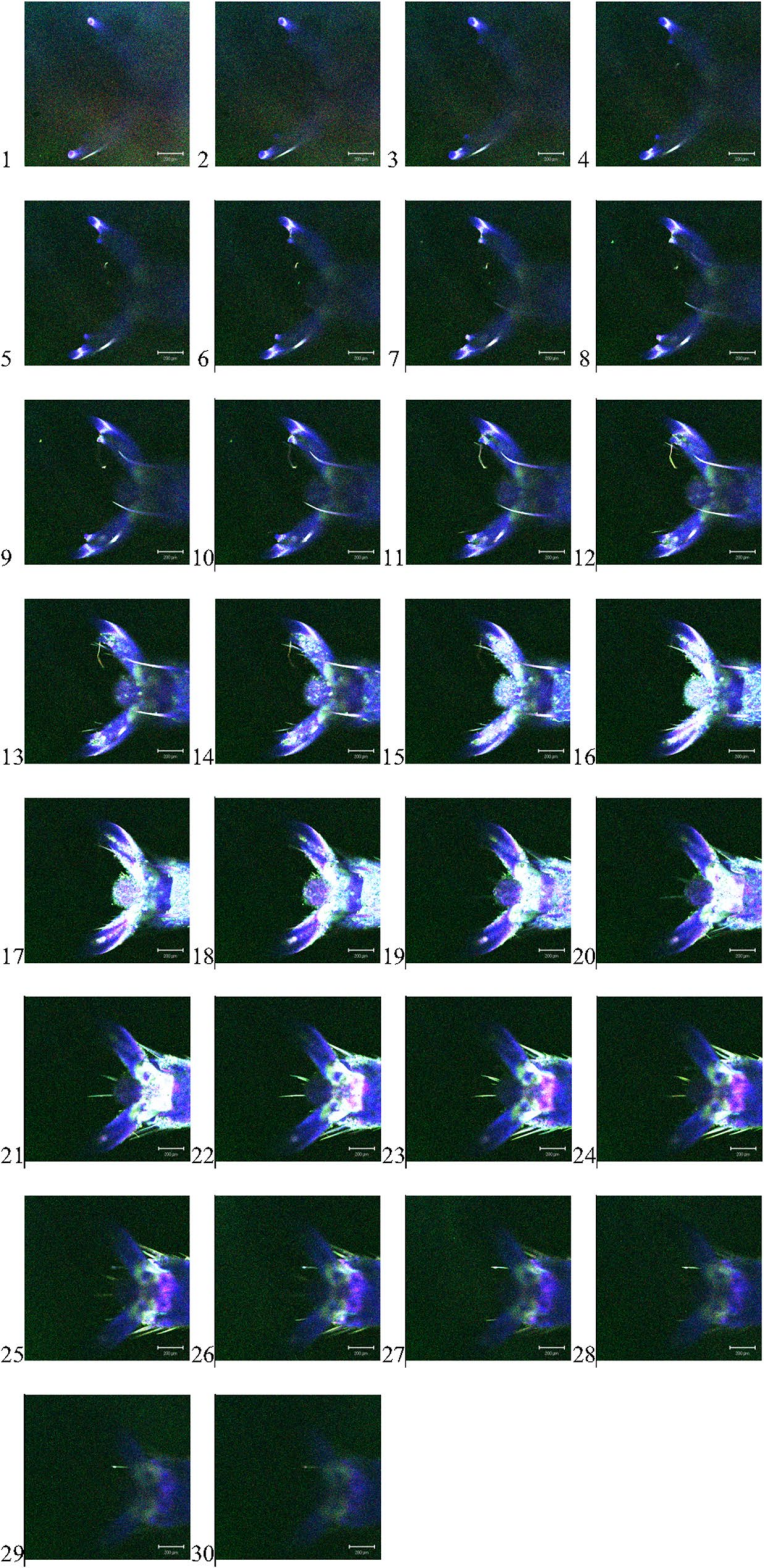
*1*–*30* The individual z-axis scan.tif files of the scan through the tarsus of a common grasshopper (the *scale bar* defines 200 μm. Dr I Jones October 2002)

Figures 3 and 4 show the compound tarsal structures of two insects, a common grasshopper and a common bee, both composed of insect chitin. Tarsi and setae are clearly visible. The structure and function of insects have been studied extensively [7].

### Common British green grasshopper tarsal structures .tif images

It is observed that there are two hooked claws to the walking structure that must interfere in some way with the substrate to produce a ground turbulent like effect such that is necessary to do finite element analysis on the posture of said structure. Gate is the most common effect of the entire school of thought with regards to insect tarsi but in fact it is a probabilistic fastener like the burdock of the product now known as Velcro that could be suited to robotic clasping mechanisms or some such structure that maintained a machine control over its structures, in handling.

Therefore, it is important to preserve its nature so that it can be studied and reproduced via inorganic or organic means for the purposes of micro surgery, visual reality and insecticide problems with genetics.

### Common british honeybee tarsal structure .tif images

It is shown here that the arolium is interacting with the two claws as it gains a foothold on the surface. Again it is important that we understand the efficacy of the bee population if we are to gain another glimpse of the wain of the sound of the fall of the migrant pine tree in the winds of chiming suns. In other words it is of use only to the known predators of the bee and its new neighbours, us, who seek to abstain from this dissipation from life as we know it but they do disappear too you see? So we must protect against the environment from being assailed by the new time called the age of the dawn of the endangered species as foodstuffs such as the bee’s pollen gathering produces, a ground turbulent like effect and with it, our appetite for it.

## Discussion

The successful imaging of the two biomaterial specimens in simply mounted slides shows that it should be possible to accumulate a library of data of samplings of these material micro-structures and frictional devices and thereby extrapolate to an even further degree to produce those needed shapes that require a sensitive beginning like self-assembly.

To have self-assembly without life is a strange concept yet it is with us already. The control system that dictates the layers is complex yet it does so.

The success of the experiment depended upon capturing the.stl file of the shapes, all three of them. This was not done so it fails. But it does illustrate the possibility at large for a further increase in the rate of research into micro-robotics and nano/self-assembled material structures [8] and their thought-provoking tendency to be annoyingly under-estimated.

## Conclusions

Confocal microscopy is commonly used in the field of neuroscience. Its application to the field of biomimetic study could be controversial since it is expensive hardware and memory intensive. This notwithstanding, memory capacities are increasing and technology advancing at a speed which may make its use more frequent in the future.

In reality a confocal microscope and its output is a laser scanner like many others on the market, engineered for microscopic applications. The random nature of measurement of light wavelengths is not suitable for conversion to .stl file and as of this moment has not been found. In order to proceed with the biomimetic process of developing a product that had the same or approximated the characteristics as the *A. minus* hook or of the chitin tarsi then we have need to consider the options open to us. Reflecting upon our need for CAD/CAM systems it becomes surmountable to be able to nurture the growth of these micro-organisms or organelles through our attempt at life-creation through self-assembly. In the meantime if one asks oneself for real reason to produce something it is to be sold and to create a market is not an impossible thing, to sell whatever you like to manufacture. So one begins by asking is this going to be the real produced product and the answer is no and, therefore, the test proceeds at a pace set for this.

It proceeds now with the acquiring of the sought after .stl file that is required for the production of a resin model in a layered manufacturing laboratory.

The generation of a biomimetic product from a biological structure combines prescriptive and descriptive processes. At present technology does not allow us to make the transition smoothly. Finite element analysis and graphical representation (virtual reality software) utilise the 3-D meshing of data points for differing purposes.

Small biological structures of cellulose and insect chitin are translucent to laser light and, therefore, can be scanned with non-destructive results. This may also be true of other biomaterials. Commercially available software can be utilised to convert the resultant data to 3-D models but this does not mean that the model is suitable for mechanical analysis.

In terms of efficiency based upon cost and computational effectiveness including memory storage, confocal microscopy is more expensive than conventional methods of sectioning and digitising. However, in terms of operator time it is by far the cheapest.

It is yet to be proved possible to use the confocal microscope to perform data capture and export the product to a finite element package, thereafter to re-mesh to perform finite element analysis.

It has been established in part 1 that the burdock hook consists of a uniform biomaterial which is anisotropic. It exhibits scaling effects, being stronger than indicated by Newtonian modelling.

This is the property that must be emphasised as it is this that would give a potential useful structure a reason for being and derive a desire to reproduce it in a controlled fashion a la mass production.

Given the applications of this technology it must be said that the end of the silence is near as we begin to delve into the world of soft tissue reproductions of living things as we develop new materials.

Many surface structures and arresting mechanisms are frictional in nature and this too is a scaled effect to be taken into account or to be applied. They are also non-homogenous and utilise differing qualities to achieve their end goal, be it adhesive by glues, anchorage by hooking or physical interference of structures or probabilistic frictional fastening for instance. Often taking the form of secondary structures themselves they are integrated into the design and function of the primary such as joint hinges, wing arrestors or the locking of two large separate body parts by sex organs by varying viscosity of interior gel-like plasm and structural membranes. These simple mechanisms are appreciated for their reduced energy usage and they are homogenous and isolated, thermodynamically speaking, within closed systems of low energy self-assembly.

To discuss the choice of design to advance further in this discourse, the three specimens all subjected to confocal microscopy are frictional devices and their physical forms are important to their function. The *A.minus* hook is suitable for fibres, already a feasible substrate for illustration of a viable frictional, probabilistic attachment mechanism that is biomimetic to *A.minus,* whereas the grasshopper and bee tarsi would be suitable for a silent probabilistic frictional attachment mechanism with further work on a suitable supplementary substrate.
